# Alpha suppression and connectivity modulations in left temporal and parietal cortices index partial awareness of words

**DOI:** 10.1016/j.neuroimage.2016.03.025

**Published:** 2016-06

**Authors:** Lorenzo Magazzini, Philipp Ruhnau, Nathan Weisz

**Affiliations:** a Cardiff University Brain Research Imaging Centre (CUBRIC), School of Psychology, Cardiff University, Cardiff, UK; b Centre for Cognitive Neuroscience, Paris Lodron Universität Salzburg, Salzburg, Austria; c Center for Mind/Brain Sciences (CIMeC), University of Trento, Trento, Italy

**Keywords:** MEG, Magnetoencephalography, NCC, Neural correlates of consciousness, GNW, Global neuronal workspace, NT, Near-threshold, CoLFIS, Corpus e Lessico di Frequenza dell'Italiano Scritto, DLP, Digital light processing, HPI, Head-position indicator, ANOVA, Analysis of variance, ERF, Event-related field, FFT, Fast Fourier transform, DICS, Dynamic imaging of coherent sources, MNI, Montreal Neurological Institute, MRI, Magnetic resonance image, CI, Confidence interval, BA, Brodmann area, VWFA, Visual word form area, TMS, Transcranial magnetic stimulation, Alpha oscillations, Functional connectivity, Magnetoencephalography, Near-threshold, Visual masking

## Abstract

The partial awareness hypothesis is a theoretical proposal that recently provided a reconciling solution to graded and dichotomous accounts of consciousness. It suggests that we can become conscious of distinct properties of an object independently, ranging from low-level features to complex forms of representation. We investigated this hypothesis using classic visual word masking adapted to a near-threshold paradigm. The masking intensity was adjusted to the individual perception threshold, at which individual alphabetical letters, but not words, could be perceived in approximately half of the trials. We confined perception to a pre-lexical stage of word processing that corresponded to a clear condition of partial awareness. At this level of representation, the stimulus properties began to emerge within consciousness, yet they did not escalate to full stimulus awareness. In other words, participants were able to perceive individual letters, while remaining unaware of the whole letter strings presented. Cortical activity measured with MEG was compared between physically identical trials that differed in perception (perceived, not perceived). We found that compared to no awareness, partial awareness of words was characterized by suppression of oscillatory alpha power in left temporal and parietal cortices. The analysis of functional connectivity with seeds based on the power effect in these two regions revealed sparse connections for the parietal seed, and strong connections between the temporal seed and other regions of the language network. We suggest that the engagement of language regions indexed by alpha power suppression is responsible for establishing and maintaining conscious representations of individual pre-lexical units.

## Highlights

• Near-threshold visual masking is used to characterize partial awareness of words.
• Partial awareness is indexed by left temporal and parietal alpha power suppression.
• Functional connectivity dissociates nodes in temporal and parietal cortices.

## Introduction

How conscious perception relates to sensory processing in the human brain is a major unanswered question in cognitive neuroscience. In recent years, various influential theoretical frameworks have stimulated a lot of experimental work to identify the neural correlates of consciousness (NCC; Crick and Koch, 1998). One prominent theory, the global neuronal workspace (GNW; Baars, 2005, Dehaene et al., 1998), proposed that a stimulus can be consciously perceived when, after activating essential nodes in sensory regions, the signal is distributed globally in the cortex and reverberates among high-level cortical areas, including frontal, temporal and parietal regions (Dehaene et al., 2001, Dehaene and Changeux, 2005, Nakamura et al., 2005). Crucially, neuronal activity confined to specific early sensory areas would thus not suffice for conscious perception.

Interestingly, Kouider et al. (2010) recently suggested that perception of a stimulus can result in different levels of conscious representation. According to the partial awareness hypothesis (Kouider et al., 2010, Kouider and Dupoux, 2004), objects are not necessarily perceived in an all-or-none manner, but rather we could be aware of certain object features while being unaware of others (see also Cleeremans, 2007, Overgaard et al., 2006, Sergent and Dehaene, 2004, on the debate of whether consciousness is a graded or dichotomous phenomenon). Therefore, we could become conscious of different properties of an object, organized hierarchically from low-level to complex forms of representation, independently.

To investigate this hypothesis, we developed a paradigm that exploits the strengths of two different approaches in the tradition of consciousness research (Dehaene et al., 2006), namely near-threshold (NT) and visual masking paradigms. The outstanding value of NT paradigms consists of providing researchers with the opportunity to isolate the neuronal processes that reflect qualitative changes in perception. The opportunity is achieved by presenting physically identical stimuli at the individual perceptual threshold, which results in stimuli being consciously perceived in only a proportion of trials. However, as a consequence of the threshold-level reduction of stimulus intensity, NT paradigms can hardly preserve the more fine-grained features of complex stimuli. To circumvent this problem, visual masking techniques have been extensively employed (see Breitmeyer and Ogmen, 2000, Enns and Di Lollo, 2000, Kouider and Dehaene, 2007 for reviews), particularly in the study of language processing (see Dehaene et al., 2001 for an example). Conventionally, the strength of the mask interference is changed to make stimuli either completely visible or completely invisible, thereby retaining all or none of their characteristics into consciousness.

We designed a word masking paradigm in which the mask's strength was adjusted to a constant threshold level, similar to the NT approach. In agreement with the partial awareness hypothesis, we defined our threshold as the chance-level probability of detecting letters in masked word presentations. Hence, we prevented participants from perceiving the entire word, and expected them to report seeing letters in only a proportion of trials. Our manipulation provided us with an ideal scenario to study the neural basis of a clearly characterized condition of partial awareness.

Specifically, the perception of letters marks the transition between two distinct levels of stimulus representation. On the one hand, letters become consciously visible by virtue of combining basic geometrical shapes into meaningful patterns. On the other hand, neuronal activation in response to letter patterns is not yet sufficient to trigger full stimulus awareness, and perception does not escalate to accessing the lexical and semantic properties of the word. In line with the GNW model outlined above, we predicted that partial awareness of words would be reflected by the engagement of core language-processing regions, also referred to as essential nodes (Zeki and Bartels, 1999; see Tong et al., 1998 for an example). To investigate this, we compared neuronal oscillatory activity, measured with MEG, between perceived and not perceived stimuli. We hypothesized that differences in oscillatory power would be found in cortical regions of the left hemisphere that represent the stimulus features disentangled by our experimental manipulation. Based on the anatomical locations showing the greatest power effects, we then used a seeded functional connectivity analysis approach to explore the functional interactions between nodes of the language network.

## Materials and methods

### Participants

Twenty right-handed healthy volunteers (mean age ± standard deviation: 25 ± 5.2; ten females) took part in the study after giving informed consent. Following behavioral data analysis, three participants were excluded due to inadequate task performance (two of them because of unbalanced proportion of perceived and not perceived stimuli, and one of them because of too many false alarms, see Behavioral data analysis below). Therefore, the analysis of MEG data was performed on seventeen out of the initial twenty participants. All participants were native Italian speakers and, to prevent confounding effects in the lexical decision task, were required to have no to very poor knowledge of the Spanish language. All participants had normal or corrected-to-normal vision and reported no known history of neurological or psychiatric disorders. The experimental procedure was approved by the local ethics committee and participants received monetary reimbursement for their participation.

### Stimuli and procedure

The experiment consisted of the adaptation of a visual masking paradigm, in which masked linguistic stimuli were presented at the individual threshold for the detection of letters, pre-estimated with two independent one-up one-down staircases converging to 50% detection rate. The visibility of the letters was manipulated by adjusting the luminance contrast of the masks. Prior to beginning the main experiment, participants completed 20 training trials that served to test if the contrast threshold had been estimated successfully. Two different sets of stimuli (each including both words and pseudowords) were used for the threshold and training procedures, none of which was presented in the main experiment. In the main experiment, participants had to perform two sequential tasks. Firstly, they had to report whether they were able to identify at least one letter in the stimulus presentation. This constituted the perceptual decision, or detection task. Secondly, they were required to perform a lexical decision and classify the stimulus as word or pseudoword. Participants were instructed to also respond when the stimulus was not perceived, i.e. irrespectively of whether they had perceived letters or not. The total time of the experimental session, including preparation, was less than 2 h.

**Fig. 1:**
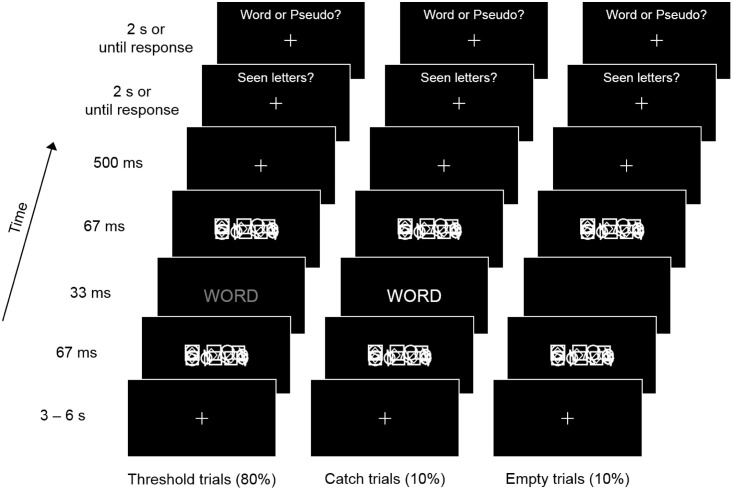
Trial structure of the experimental paradigm. Each trial started with a fixation cross for a jittered period of 3 to 6 s. The stimulation sequence consisted of the consecutive presentation of a forward mask (67 ms), followed by a four-letter stimulus (33 ms) and by a backward mask (67 ms). The color of the masks was adjusted individually with a staircase procedure prior to the experiment. The letters constituted either a word or a pseudoword, and were presented in gray (trials at threshold), in white (catch trials, increased visibility), or in black (empty trials, invisible). After an interval of 500 ms, participants were required first to report if they had perceived any letters (detection task) and second to classify the stimulus as word or pseudoword (lexical decision, or classification task).

The trial structure of the experiment is illustrated in Fig. 1. Target stimuli were presented with three different intensities (320 trials at threshold, 40 supra-threshold catch trials and 40 empty trials), in random order and equally distributed across eight blocks (~ 5 min each). Participants were allowed to take breaks between blocks. Target stimuli consisted of strings of four letters forming either a word or a pseudoword (two stimulus classes, see Stimuli and procedure below). In threshold trials, letters were presented in medium-contrast gray. In catch trials, letters were presented in white to increase their visibility. In empty trials, letters were presented in black and were therefore invisible. Each trial began with a white fixation cross (0.8 × 0.7° visual angle) presented centrally on a black background for a jittered period of 3 to 6 s, with shorter intervals being more common than longer ones. The target stimulus was presented centrally (3 × 1° visual angle) for 33 ms, and was immediately preceded and followed by a forward and a backward mask of 67 ms duration each. Masks consisted of a fixed number of different shapes (squares, diamonds, triangles and circles) drawn with the same line thickness as the text font (Arial) and covering the same approximate area. The size and position of the shapes was pseudo-randomly generated on a trial-by-trial basis, but with forward and backward masks of the same trial remaining identical. The fixation cross disappeared at forward mask onset and reappeared at backward mask offset. After a 500 ms interval, two questions were displayed consecutively on the screen for up to 2 s each, or until a response was given. Participants responded ‘perceived’ or ‘not perceived’ to detection and ‘word’ or ‘pseudoword’ to classification by raising the index or middle finger of their right hand. The responses associated with the index and the middle finger were counterbalanced in all possible combinations, both across participants and between the first and second response.

The two classes of target stimuli consisted of words and pseudowords, equally distributed across levels of stimulus intensity (threshold, catch, and empty trials) and experimental blocks. Words were selected from the CoLFIS database (Bertinetto et al., 2005) and consisted of Italian disyllabic nouns only, syllables having consonant–vowel structure. Pseudowords were created by substituting the second consonant of each word stimulus, following the phonotactic restrictions of the Italian language. Pseudowords that were English words and pseudowords that would become Italian words by adding an accent to the final vowel were excluded. Words and pseudowords did not differ statistically in bigram frequency (*t*_(398__)_ = 1.28, *p* = 0.2) and neighborhood size (*t*_(398)_ = 1.02, *p* = 0.3).

All stimulus presentations were programmed in Matlab (MathWorks) using the Psychophysics Toolbox (Kleiner et al., 2007). Stimuli were back-projected onto a translucent screen using a DLP projector (Panasonic PT-D7700E) located outside the magnetically shielded room. The projected area was 17 × 13.6° visual angles (with a resolution of 1280 × 1024 pixels) and the refresh rate was 60 Hz. A photodiode was placed at the upper left corner of the projection screen in order to record the exact onset of the visual stimulation.

### Data collection

Whole-head recordings were performed using a 306-channel (102 triplets composed of one magnetometer and two planar gradiometers) MEG system (Neuromag306; Elekta) in a magnetically shielded room. The sampling rate was 1000 Hz and hardware filters were used to band-pass the measured signal between 0.1 and 330 Hz. Prior to the MEG recording, the location of five head-position indicators (HPI coils) and a minimum of 200 head-shape samples were digitized (3Space Fastrack; Polhemus) relative to three anatomical landmarks on the participant's head (nasion and left and right pre-auricular points). The head position inside the MEG helmet was localized at the beginning of each experimental block for co-registration purposes.

### Behavioral data analysis

Signal detection theory was used to test participants' ability to discriminate a stimulus presented at threshold from background noise (empty trials). For this purpose, we defined perceived threshold trials as ‘hits’, not perceived threshold trials as ‘misses’, perceived empty trials as ‘false alarms’, and not perceived empty trials as ‘correct rejections’. The *d′* sensitivity measure was calculated for each participant, and the group performance was tested with a one-sample *t*-test against a sample mean of 0 (representing chance-level performance). To test whether trials at threshold were more likely to be reported as perceived or not perceived, detection rates were analyzed with a one-sample *t*-test against a sample mean of 0.5.

For the analysis of the classification task, firstly, we obtained a measure of classification bias. This indexed participants' likelihood to make either one of the classification responses (word or pseudoword), irrespective of classification accuracy. Secondly, we sorted trials according to stimulus class (word or pseudoword), and tested participants' ability to classify words and pseudowords as such. The ratio of correct classifications was calculated separately for each of the four possible combinations of stimulus class and stimulus intensity, i.e. threshold words, threshold pseudowords, catch words, and catch pseudowords (not for empty trials, as stimuli presented in this condition were completely invisible). Finally, we sorted trials according to stimulus detection (perceived and not perceived), and tested whether participants' ability to perform the classification task varied depending on whether the stimulus was perceived or not. The ratio of correct classifications was calculated separately for each of the four possible combinations of stimulus detection and stimulus intensity, i.e. threshold perceived, threshold not perceived, catch perceived, and catch not perceived. Statistical analyses were performed by means of one-sample *t*-tests against a sample mean of 0.5.

Response times to the first and second questions in trials at threshold were calculated separately for each of the four possible combinations of stimulus class (word and pseudoword) and stimulus detection (perceived and not perceived). Statistical analysis was performed using a two-way repeated measures analysis of variance (ANOVA) with factors ‘task’ (2 levels: detection and classification) and ‘stimulus’ (4 levels: perceived word, not perceived word, perceived pseudoword, and not perceived pseudoword).

### MEG data analysis

The analysis was performed using Matlab, the FieldTrip toolbox (Oostenveld et al., 2011) and SPM8 (Litvak et al., 2011). The MEG data were epoched (± 3 s locked to target stimulus onset), high-pass filtered at 1 Hz using a two-pass, 6th order Butterworth filter, and down-sampled to 250 Hz. Epochs were concatenated across blocks and inspected for artifacts using a semi-automatic procedure. Trials that contained eye blinks or muscle activity and sensors that were consistently contaminated by artifacts (e.g. channel jumps) were identified and removed from the dataset. The rejected channels were interpolated using the nearest-neighbor approach and used for analysis of sensor-level, but not source-level, data.

#### Event-related fields analysis

Prior to event-related fields (ERFs) calculation, data were band-pass filtered between 2 and 20 Hz using a two-pass, 4th order Butterworth filter. Trials were baseline corrected by subtracting the mean activity in a − 0.3 to − 0.1 s time-interval preceding target stimulus onset from the whole individual trial, and then averaged. For planar gradiometers, the combined amplitude was computed at each orthogonal gradient pair according to Pythagora's theorem.

#### Time–frequency analysis

Frequency-domain analysis was performed for each trial separately. Spectral estimates were computed using fast Fourier transform (FFT) with a single Hanning taper, for time windows of 500 ms (from − 1 to 1 s in steps of 50 ms) and frequencies between 4 and 34 Hz (in steps of 2 Hz). Power values were calculated separately for the vertical and horizontal component of the planar gradients, and combined via their sum. The resultant frequency solutions were averaged across trials, resulting in total power.

#### Statistical analysis

Statistical analyses of ERFs and time–frequency representations at sensor level were performed using a non-parametric cluster-based permutation test. By increasing the sensitivity of the statistical test, while controlling for the multiple comparison problem, this approach is particularly advantageous for statistical MEG data analysis (Maris and Oostenveld, 2007). Firstly, clusters of statistically significant differences (paired-sample *t*-tests between conditions, exceeding the threshold of *p* < 0.05) that were adjacent in space, time, and frequency were identified. Secondly, the *t* statistics within each cluster were summed up, and a cluster-level maximum statistic was obtained by selecting the cluster with largest sum statistic (both for positive and negative clusters). Thirdly, 10,000 random permutations of the data (data exchanged between conditions within the participants) were generated using the Monte Carlo method, and a reference distribution of maximum statistics was produced under the null hypothesis that the data were not different between conditions. Finally, the *p*-value of the cluster-level statistic observed on the data was estimated from the proportion of values in the reference distribution exceeding the observed maximum statistic (and then Bonferroni-corrected for the probability of observing both positive and negative significant clusters).

#### Source analysis

To identify the probable generators of the time–frequency effect observed at sensor level (see Induced responses below), source localization was performed using a frequency-domain beamforming algorithm (dynamic imaging of coherent sources, DICS; Gross et al., 2001). For this purpose, the canonical cortical anatomy of each participant was generated via affine transformation of a Montreal Neurological Institute (MNI) template onto the subject's digitized head shape. The pseudo-individual MRI was then divided into a grid of equidistant points (1 cm resolution) and a single-shell head model (Nolte, 2003) was used to compute the leadfield at each grid location. The cross-spectral density matrix was estimated using multitaper FFT with Slepian tapers, within the time–frequency window of the effect revealed by the sensor-level analysis (12 ± 3 Hz, 0.5–1 s, see also Induced responses below). A common spatial filter was computed for each grid location using data epochs from all trial conditions. Source power was then estimated in each condition by applying the beamformer filter to each condition separately.

#### Source-level connectivity analysis

Using an exploratory whole brain approach we investigated connectivity patterns based on seeds determined by the source-level power effect. To do this we computed source-level Fourier coefficients by first estimating the cross-spectral density in a time–frequency window centered on the sensor-level effects (12 ± 4 Hz, 0.5–1 s). Secondly, we computed beamformer filters (Gross et al., 2001) from the combined data sets (perceived and not perceived) using the leadfields for the equidistant grid as described above. These filters were then applied to sensor-level Fourier coefficients, which were estimated using multitaper FFT with Slepian tapers (12 ± 4 Hz, 0.5–1 s). Finally, the source-level Fourier coefficients served to estimate the cross-spectral density on source level, from which we calculated coherence, a measure of phase synchrony between grid point derived from the complex part of the cross-spectrum (Nolte et al., 2004). We evaluated differences in coherence between perceived and not perceived stimuli by contrasting the values for the two grid points with the maximum power effect in the parietal [MNI coordinates: − 32 − 54 41 mm] and temporal cortex [− 62 − 57 11 mm] with paired-sample *t*-tests, thresholded for *p* < 0.01.

## Results

### Behavioral results

**Fig. 2:**
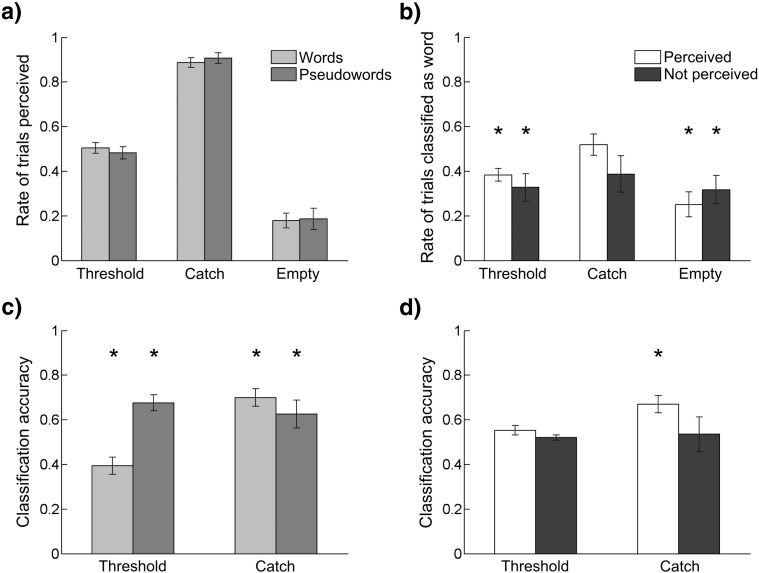
Behavioral results. a) Detection rate, i.e. rate of threshold, catch, and empty trials that were reported as perceived in the detection task, plotted separately for words and pseudowords. b) Classification bias, i.e. rate of threshold, catch, and empty trials that were classified as words, irrespective of classification accuracy, plotted separately for perceived and not perceived stimuli. c) Classification accuracy of words and pseudowords, irrespective of stimuli being perceived or not. Note that statistical significance in trials at threshold is a spurious result of the classification bias shown in b), see also Behavioral results. d) Classification accuracy of perceived and not perceived stimuli, irrespective of stimuli being words or pseudowords. Results of statistical analyses are shown in panels b), c), and d), with stars indicating significant results (*p* < 0.05) of one-sample *t*-tests against a sample mean of 0.5. Error bars represent standard error of the mean. Words are represented in light gray, pseudowords in dark gray, perceived stimuli in white, and not perceived stimuli in black.

The analysis of the detection task indicated that our experimental manipulation of letter-visibility was successful. The rate of stimuli reported as perceived was 0.5 for trials at threshold (95% CI [0.43, 0.57]), 0.9 for catch trials (95% CI [0.85, 0.94]), and 0.2 for empty trials (95% CI [0.12, 0.33]), with similar detection rates for word and pseudoword stimuli (Fig. 2a). The signal detection theory analysis confirmed that participants were more likely to perceive stimuli presented at threshold, compared to incorrectly reporting empty trials as perceived (*d′* = 0.95, *t*_(19)_ = 7.3, *p* < 0.001). The probability of reporting threshold trials as perceived or not perceived was at chance level (*t*_(19)_ = 0.1, *p* = 0.9).

The analysis of classification bias (Fig. 2b) revealed that, irrespective of whether stimuli were perceived or not, participants were more likely to respond ‘pseudoword’ in the classification task when stimuli were presented at threshold (perceived, *t*_(19)_ = − 4.5, *p* < 0.001; not perceived, *t*_(19)_ = − 3.6, *p* = 0.002), or in empty trials (perceived, *t*_(19)_ = − 4.2, *p* < 0.001; not perceived, *t*_(19)_ = − 3.8, *p* = 0.001), but not in catch trials (perceived, *t*_(19)_ = 0.4, *p* = 0.7; not perceived, *t*_(19)_ = − 1.7, *p* = 0.1). This caused the accuracy rates of words and pseudowords at threshold (Fig. 2c) to be significantly different from chance level (words, *t*_(19)_ = − 3.5, *p* = 0.002; pseudowords, *t*_(19)_ = 5.4, *p* < 0.001), a spurious result of the classification bias. The classification accuracy of catch trials (Fig. 2c), which were not affected by any bias in classification, was significantly better than chance both when the stimulus was a word (*t*_(19)_ = 5.2, *p* < 0.001), and when it was a pseudoword (*t*_(19)_ = 2.6, *p* = 0.02). This indicates that, irrespective of whether they were perceived or not, words and pseudowords were classified with better than chance accuracy when they were presented with supra-threshold intensity.

Participants' ability to correctly discriminate between words and pseudowords, i.e. classification accuracy, is shown in Fig. 2d as a function of stimulus detection (separately for perceived and not perceived stimuli). When stimuli were presented at threshold, classification accuracy was not significantly better than chance level. Although the rates of correct classification approached statistical significance (perceived, *t*_(19)_ = 2.0, *p* = 0.058; not perceived, *t*_(19)_ = 1.4, *p* = 0.17), the size of the effect was small (perceived, 95% CI [0.49 0.58]; not perceived, 95% CI [0.49 0.54]). When stimuli were presented supra-threshold, i.e. in catch trials, classification accuracy was significantly better than chance when the stimulus was perceived (*t*_(19)_ = 4.8, *p* < 0.001), but not when the stimulus was not perceived (*t*_(19)_ = 0.9, *p* = 0.4). This indicates that stimuli, irrespective of being words or pseudowords, were classified with better than chance accuracy only if they were presented with supra-threshold intensity and perceived.

The results of ANOVA on response times showed no effect of task (*F*_(1,19)_ = 2.6, *p* = 0.1) or stimulus (*F*_(3,57)_ = 0.3, *p* = 0.8) and no interaction effect (*F*_(3,57)_ = 1.7, *p* = 0.2). Therefore, in trials at threshold, there were no differences in participants' detection and classification response times, irrespective of stimuli being words, pseudowords, perceived, or not perceived.

### MEG results

#### Evoked responses

Non-parametric cluster-based statistical testing of ERFs did not reveal a significant difference between perceived and not perceived stimuli at threshold. Using a time window of − 0.3 to 0.5 s relative to target stimulus onset, clusters of statistically significant differences (*α* = 0.05) were not observed for either type of sensors (magnetometers and combined planar gradiometers). This indicates that stimuli presented at threshold did not result in evoked responses of different amplitude depending on whether they were perceived or not.

#### Induced responses

Statistical testing of sensor-level time–frequency representations for perceived and not perceived stimuli at threshold was performed over the entire frequency range, in two distinct time windows. In a time window of − 1 to 0 s, the observed clusters were not significant (*p* > 0.05). In a time window of 0 to 1 s, the analysis revealed one significant cluster (*p* = 0.0005), indicating a statistically significant difference between the two conditions in this time window. Therefore, while no difference in power was observed for ongoing pre-stimulus oscillations, stimuli at threshold resulted in induced responses of different power depending on whether they were perceived or not perceived.

**Fig. 3:**
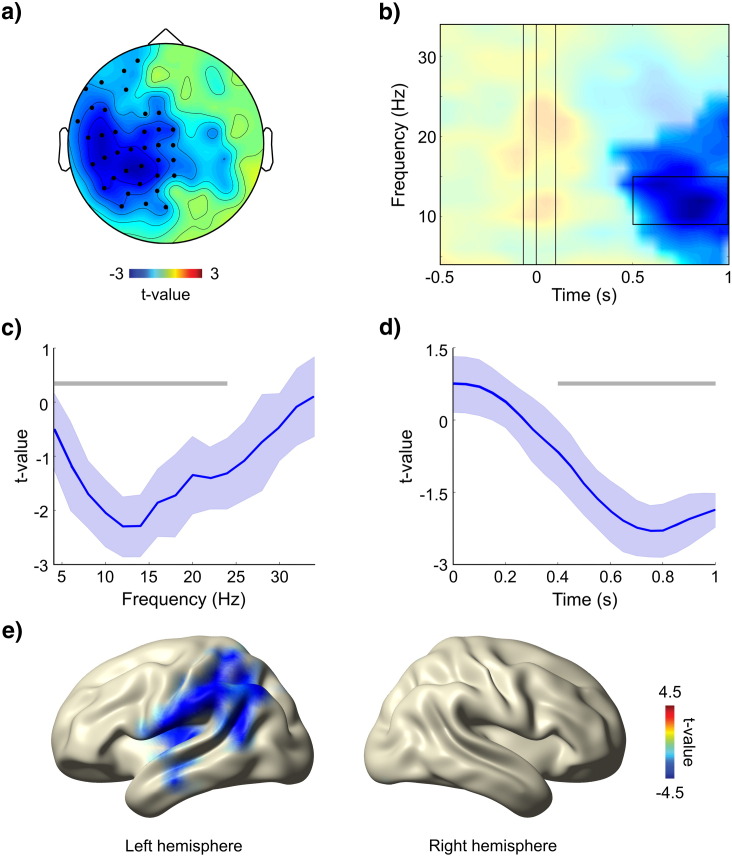
Sensor-level contrast of perceived versus not perceived stimuli at threshold. a) Scalp-level topography highlighting the cluster of sensors (*p* < 0.01) selected for sensor-level analysis. The overlaid topographical map illustrates the statistical contrast perceived versus not perceived (*t* values masked at *p* < 0.05; corrected for multiple comparisons) averaged over time (0.5 to 1 s) and frequency (8 to 16 Hz). Blue colors index lower power for perceived stimuli. b) Time–frequency representation of *t* values (masked at *p* < 0.05; corrected for multiple comparisons) averaged over the cluster of sensors shown in a). The solid black line marks the time–frequency window selected for source analysis. Color scale as in a). c) *T* values averaged over time (0.5 to 1 s), separately for each frequency bin. The horizontal gray bar illustrates the size of the cluster in frequency (*p* < 0.05). The shaded area in light-blue represents standard deviation across the cluster of sensors. d) *T* values averaged over frequency (8 to 16 Hz), separately for each time point. The horizontal gray bar illustrates the size of the cluster in time (*p* < 0.05). Shaded area as in c). e) The color-map illustrates the statistical contrast perceived versus not perceived (*t* values masked at *p* < 0.01; uncorrected) of source-reconstructed power in a time–frequency window of 0.5–1 s and 10 ± 3 Hz, overlaid on a standard MNI brain. Blue colors indicate greater decrease in alpha power for perceived compared to not perceived stimuli.

To focus our later source analysis appropriately, we identified the time and frequency windows representing the strongest sensor-level effect. For this purpose, we selected a subset of sensors corresponding to the topographically most robust effect (see highlighted sensors in Fig. 3a). The selection was performed by repeating the cluster-based permutation with more conservative criteria (*α* = 0.01 for both first and second order statistics). This left 37 of the initial 72 sensors that formed the cluster observed with *α* = 0.05. This subset of sensors was averaged, producing a time–frequency representation of averaged *t* values. As illustrated in Fig. 3b, perceived stimuli were characterized by significantly lower power in the post-stimulus period, compared to not perceived stimuli at threshold. The cluster (*α* = 0.05) was comprised of a relatively broad range of frequencies (from 4 to 24 Hz), with the greatest difference centered in the alpha range (at ~ 12 Hz; Fig. 3c). The cluster emerged at ~ 0.4 s and, in time, the greatest difference was observed at ~ 0.8 s (Fig. 3d). In summary, perceived stimuli at threshold induced a stronger decrease in alpha power, compared to not perceived stimuli at threshold. This response was sustained until at least 1 s after the onset of the stimulus, and spread over left-lateralized scalp locations, including mainly central, parietal, and temporal sensors.

#### Source localization

Using a beamformer analysis (see Source analysis above), we estimated the probable generators of the sensor-level effect between 0.5 and 1 s, for frequencies between 9 and 15 Hz (Fig. 3b). Source-power estimates for the two conditions were compared statistically by means of *t*-tests (*p* < 0.01, uncorrected). Results revealed a significantly stronger decrease in alpha power over parietal and temporal regions of the left hemisphere for perceived, compared to not perceived stimuli at threshold (Fig. 3e). The peak of activity was localized to the angular gyrus (Brodmann area [BA] 39) in the left inferior parietal lobule, and extended mainly anteriorly to the supramarginal gyrus (BA40), but also to perisylvian and superior temporal cortices (e.g. BA22), and to the middle temporal gyrus (BA21).

#### Source-level connectivity effects

**Fig. 4:**
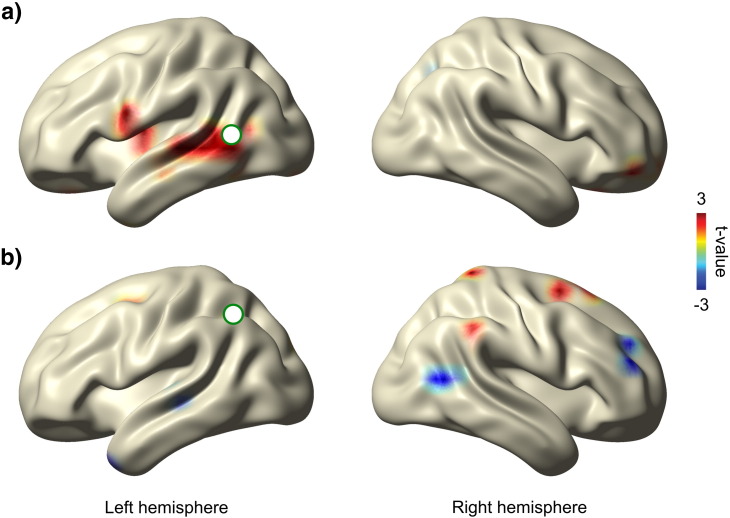
Seeded connectivity analysis. Contrasts of coherence of perceived and not perceived stimuli. The seeds (green circles) were chosen based on the power effect (see Fig. 3e). a) Coherence based on a temporal seed [− 62 − 57 11 mm] and b) based on a parietal seed [MNI coordinates: − 32 − 54 41 mm]. The *t* values are masked at *p* < 0.01 (uncorrected).

Contrasts of coherence between perceived and not perceived stimuli are shown in Fig. 4. The seed in the left temporal cortex (Fig. 4a, [− 62 − 57 11 mm]) showed stronger connections to the inferior frontal cortex and to an area covering the middle to superior temporal gyrus (BA21, 22). For the seed in left parietal cortex (Fig. 4b, [− 32 54 11 mm]) we found increased coherence for perceived compared to not perceived stimuli in a scattered pattern of contralateral temporal, parietal, and frontal areas.

## Discussion

In the present study, we investigated the partial awareness hypothesis (Kouider et al., 2010), which states distinct features of a stimulus can be consciously represented independently of each other. To test this hypothesis we used visually presented words and pseudowords. These stimuli can be broken into a number of distinct features, providing an ideal scenario for conscious perception to arise at partial levels of representation. Importantly, each of these representational levels can be mapped onto a distinct process of visual word recognition, including visual feature analysis, orthographic processing of letters and letter strings, grapheme-to-phoneme conversion, lexical access and semantic knowledge retrieval (see Carreiras et al., 2014 for a comparison of modular and interactive accounts of these processes). Here, we disentangled a pre-lexical stage of visual word recognition consisting of the orthographic representation of single alphabetic letters, the building blocks of words. By isolating this representational level, we identified a clear condition of partial awareness in which the properties of the stimulus begin to emerge but are not yet fully represented within consciousness.

This was achieved with a visual word masking paradigm, in which the masks were adjusted individually and resulted in perception of letters in approximately half of the trials. The low-level properties of words and pseudowords were carefully controlled for, and performance in a lexical decision task was monitored to ensure that perception did not escalate to full stimulus awareness. Thus, by virtue of our experimental manipulation, participants could only perceive individual letters, while remaining unaware of the whole letter strings presented. Crucially, the mask intensity was identical across trials, thereby preventing changes in perceptual awareness resulting from differences in stimulation. We hypothesized that perceiving letters consciously would depend on cortical activity in essential nodes that support pre-lexical orthographic processing in visual word recognition. We found that the transition from absence of awareness to partial awareness of words was marked by suppression of oscillatory alpha power in temporal and parietal regions of the left hemisphere. We suggest that this result reflects the engagement of cortical areas that are responsible for establishing and maintaining conscious representations of individual pre-lexical units.

The neuronal bases of visual word recognition (in this context also simply referred to as ‘reading’) lie in a network of cortical regions lateralized to the dominant, typically left, hemisphere. According to the classic dual-route account (Coltheart et al., 1993; see Price, 2012, Pugh et al., 2000a for reviews, and Jobard et al., 2003 for a meta-analysis), this network consists of two separate processing streams, a dorsal pathway (including the left supramarginal and angular gyri, as well as the superior temporal gyrus), and a ventral pathway (comprising mainly left inferior occipito-temporal regions). In visual word recognition paradigms, both pathways have been imaged with MEG using source-reconstruction of 10–20 Hz oscillatory activity (Cornelissen et al., 2009, Pammer et al., 2004, Pammer et al., 2006). Consistent with these studies, the suppression of oscillatory power that we observed at sensor level peaked in the alpha range (~ 12 Hz) and spanned beta-band frequencies up to approximately 24 Hz. The alpha suppression was particularly pronounced in the left angular and supramarginal gyri. These regions overlap with only part of the more extended network involved in reading whole words, indicating that conscious representations at the level of single letters were supported mainly by the dorsal pathway. To explore the different contribution of temporal and parietal regions within this pathway, we performed a seeded functional connectivity analysis. Interestingly, while the left parietal seed showed scattered connections distributed over the right hemisphere, the connectivity pattern of the left temporal seed was more consistent, with strong connections between left temporal and left frontal regions of the dorsal network.

The observation that only a subset of essential nodes appeared to be specifically activated under conditions of partial awareness suggests that our design was effective in confining visual word recognition to the mere process of letter identification. Recently, it has been proposed that the manner in which orthographic processing interacts with phonological and semantic information can be shaped differently in different languages (Frost, 2012). Specifically, Italian orthography is consistent, i.e. based on simple grapho-phonological conversion rules, whereas English orthography is inconsistent and requires complex rules for mapping letters to sounds. Therefore, Italian graphemes can be unequivocally converted into the corresponding phonemes, and this could explain the greater engagement of dorsal regions in Italian readers, as opposed to the ventral pathway in English readers (Paulesu et al., 2000). Our study did not require participants to access specific mapping rules stored in ventral regions (Szwed et al., 2012). Therefore, the recruitment of the dorsal pathway may reflect the dependence of visual orthographic information on grapho-phonological conversion, which would allow the visual representation to become conscious despite strong visual masking.

One aspect of our results that appears important to consider is the timing of the alpha effect. According to the GNW model (see Dehaene and Changeux, 2011 for a review), conscious representations would be triggered as late as 300 ms after presentation of a stimulus, as neuronal activity reverberates among high-level cortical areas (Del Cul et al., 2007). This could explain, at least in part, why no difference was observed between the evoked responses to perceived and not perceived letters, within the first 500 ms after stimulus onset. Rather, the temporal evolution of alpha power observed here is in line with the idea of recurrent neuronal activity outlasting input termination (Fisch et al., 2009; see Lamme, 2006, Lamme and Roelfsema, 2000 for reviews). Thus, the alpha suppression could reflect local recurrent processing along the dorsal route, either by enhancement of cortical excitability (Klimesch et al., 2007) or by disengagement from functional inhibition (Jensen and Mazaheri, 2010). The GNW further predicts the neuronal signal to reverberate globally across not only temporal and parietal cortices, but also frontal association cortices. In support of this, our connectivity analysis results showed higher connectivity between left temporal and left inferior frontal regions. Although speculative, the orthographic information could be successfully represented into consciousness by accessing the corresponding phonological information stored in the supramarginal gyrus (Stoeckel et al., 2009) and superior temporal cortices (Joseph et al., 2001). In this view, forming and retaining an object into awareness, an alphabetic letter in this case, would engage a network of nodes in which the different features of such an object are represented (see Ghazanfar and Schroeder, 2006, Spence and Bayne, 2014 for multisensory accounts resembling this perspective). These features would then be incorporated into a unique conscious representation, a process that in our study appeared to be mediated by the angular gyrus, an ideal candidate for high-level multimodal integration (Binder et al., 2009, Pugh et al., 2000b, Seghier, 2013).

The work presented here represents one of the very few studies providing evidence of the electrophysiological correlates of partial awareness (as defined in Kouider et al., 2010). In the context of partial awareness, one MEG study contrasted words perceived incompletely with words perceived fully (Levy et al., 2013). The authors claimed that the transition from partial to complete visual word recognition was marked by suppression of alpha activity in regions of the ventral pathway. However, the contrast between full and partial awareness does not reflect the emergence of partial awareness per se. Rather, a state of no awareness needs to be contrasted in order to isolate partial awareness, as we did in this study. More recently, Levy et al. (2015) reported early and late gamma-band (> 40 Hz) responses to be involved in partial and full awareness of words, respectively. Because their experimental design (e.g., task methodology and language) differed from our study, results are difficult to compare. One of the discrepancies, however, immediately rekindles an ongoing debate on visual word recognition. The study by Levy et al. (2015) implicated a portion of the left fusiform gyrus that has been proposed by some authors as a functionally specialized ‘visual word form area’ (VWFA; Cohen et al., 2000, Cohen et al., 2002). The putative role of the VWFA would consist of establishing abstract representations of letter strings, although the claim of functional specificity in this region has been strongly criticized by other researchers (Price and Devlin, 2003). If inferior temporal regions were involved in orthographic processing (a view that appears to be generally accepted, cf. Dehaene and Cohen, 2011, Price and Devlin, 2011), it appears from our study that they were not crucial to form conscious representations of single letters. Evidence from neuropsychological cases is consistent with our results, and suggests that lesions to the VWFA do not completely disrupt the ability to read (Cohen et al., 2003, Cohen et al., 2004). Thus, the VWFA could rather represent a feed-forward processing region (reviewed in Carreiras et al., 2014; see also Levy et al., 2015), projecting to higher-order cortical areas where the orthographic representation would be brought into awareness.

Finally, our conclusions should be discussed in light of recent reviews on the rationale behind the NCC research (Aru et al., 2012, de Graaf et al., 2012). Specifically, a distinction between different correlates of awareness has been proposed, with some of the NCC constituting either prerequisites or consequences of consciousness, rather than actual substrates of the conscious experience itself. The NCC prerequisites (reviewed in Ruhnau et al., 2014) can be considered to reflect the neuronal ‘state’ that precedes stimulus onset, and have been often identified in either the power (e.g., Hanslmayr et al., 2007) or the phase (e.g., Busch et al., 2009) of pre-stimulus oscillations. Although phase measures were not taken into account here, we found no differences in pre-stimulus oscillatory power between perceived and not perceived stimuli. However, this is not surprising if we assume that the use of forward masks could reset the cortical state of the visual system immediately before the presentation of each target stimulus (likewise, differences in the evoked response to perceived and not perceived letters could be obscured by the strong visual evoked responses to forward and backward masks). The NCC consequences, instead, consist of the result of conscious experience, and are much more difficult to disentangle from the NCC substrates (de Graaf et al., 2012). The latency of the alpha effect observed here is consistent with an interpretation in terms of NCC consequences, although it does not rule out predictions made by both GNW and recurrent processing theories of conscious perception (Dehaene and Changeux, 2011, Lamme, 2006). Nevertheless, due to the correlational nature of our methods, the role of the angular and supramarginal gyri in supporting partial awareness of words remains to be further investigated. For example, studies adopting neuromodulatory techniques, such as TMS (see de Graaf and Sack, 2014, Ro, 2010, Tapia and Beck, 2014 for reviews), could prove extremely useful in establishing the causal relationship between neuronal activity in regions of the dorsal pathway and conscious perception of individual orthographic units.
